# Defect-Driven Thermoelectric Decoupling in Oxygen-Deficient WO*_x_* and Tungsten Magnéli Thin Films Grown by PLD: A Review

**DOI:** 10.3390/ma19153184

**Published:** 2026-07-25

**Authors:** Enza Fazio, Priscilla Pelleriti, Carmelo Corsaro, Dario Morganti, Paolo Mele

**Affiliations:** 1 Dipartimento di Scienze Matematiche ed Informatiche, Scienze Fisiche e Scienze della Terra (MIFT), Università di Messina, Viale Ferdinando Stagno d’Alcontres, 31, 98166 Messina, Italy; priscilla.pelleriti@studenti.unime.it (P.P.); carmelo.corsaro@unime.it (C.C.); 2 Dipartimento di Scienze Chimiche, Biologiche, Farmaceutiche ed Ambientali (ChiBioFarAm), Università di Messina, Viale Ferdinando Stagno d’Alcontres, 31, 98166 Messina, Italy; dario.morganti@unime.it; 3 College of Engineering, Shibaura Institute of Technology (SIT), Omiya Campus, 307 Fukasaku, Minuma-ku, Saitama City 337-8570, Saitama, Japan; pmele@shibaura-it.ac.jp

**Keywords:** transition metal oxides, thermoelectrics, decoupling strategies, pulsed laser deposition, Magnéli phases

## Abstract

This review aims to analyze defect-driven thermoelectric decoupling in pulsed laser deposition (PLD)-grown oxygen-deficient ${WO}_{x}$ and tungsten Magnéli thin films. While transition metal oxides offer a non-toxic, abundant alternative to conventional thermoelectrics, tungsten oxide stands out due to the profound impact of sub-stoichiometry on its transport properties. We systematically evaluate how ordered oxygen vacancies and crystallographic shear planes transform insulating ${WO}_{3}$ into sub-stoichiometric phases exhibiting metallic-like conductivity. Specifically, we analyze how the delocalization of *W* 5d electrons around defect-rich regions induces electronic states near the Fermi level, decoupling the Seebeck coefficient from electrical conductivity. Simultaneously, we discuss how these engineered defect networks and shear planes selectively enhance phonon scattering, drastically suppressing lattice thermal conductivity without hindering electronic transport. By establishing PLD as an effective approach for precise oxygen stoichiometry and defect architecture control, this review highlights the high-temperature potential of tungsten Magnéli phases and outlines future pathways to maximize their thermoelectric figure of merit (ZT).

## 1. Introduction

Direct conversion of waste heat into electricity via thermoelectric (TE) materials represents a pivotal technology for sustainable energy harvesting. Historically, the field has been dominated by intermetallic compounds. However, these conventional materials face severe limitations at elevated temperatures due to thermal degradation, toxic composition, and elemental scarcity (see Appendix A and Appendix B). In this scenario, transition metal oxides have emerged as a paradigm-shifting alternative, offering exceptional chemical stability, environmental benignity, and cost-effectiveness [1,2,3]. Despite over two decades of intensive research, metal oxides are still often critically reviewed through a restrictive lens that highlights their lower electrical conductivity compared to classic intermetallics. Furthermore, traditional literature frequently relies on material-by-material compilations [4,5,6]. This review departs from such conventional approaches by providing a comprehensive, mechanism-driven evaluation of transition metal oxides. In fact, optimizing their performance remains a fundamental challenge due to the rigid interdependence of the Seebeck coefficient (S), electrical conductivity (σ), and thermal conductivity (κ). The scope of this review is to elucidate how the deliberate engineering of oxygen vacancies and crystallographic shear planes can break the historical trade-offs governing the thermoelectric figure of merit. We strictly focus on thin films, deliberately excluding metal oxide powder dispersions or bulk sintered bodies. Concentrating on the thin-film configuration allows us to explore low-dimensional transport phenomena and surface/interface-driven decoupling mechanisms that are uniquely accessible in highly oriented, continuous layers, which are ideal for micro-thermoelectric devices and surface thermal management coatings.

Within this framework, sub-stoichiometric tungsten oxide (${WO}_{x}$) is isolated as the ideal platform for high-temperature oxide thermoelectrics and as a uniquely powerful and versatile platform to investigate and decouple the physical parameters involved in transport mechanisms, outperforming other widely studied TMOs such as titanates, zinc oxides, or cobaltites. While conventional oxides typically require complex extrinsic chemical doping to tune their electronic properties, often leading to lattice destabilization, the ${WO}_{x}$ system relies on an exceptional intrinsic structural flexibility. While molybdenum oxide (${MoO}_{x}$) shares closely related structural motifs, variable oxidation states, and an analogous suboxide chemistry, ${WO}_{x}$ offers fundamental physical advantages. First, tungsten suboxides exhibit significantly higher thermal stability and a higher melting/decomposition threshold compared to ${MoO}_{3-x}$ phases, which suffer from volatile sublimation at elevated temperatures. Second, from a transport perspective, the heavier atomic mass of tungsten (*W*) compared to molybdenum (Mo) introduces stronger mass-fluctuation scattering, inherently suppressing lattice thermal conductivity ($\kappa_{L}$) to a greater extent. Moreover, the more extended spatial overlap of W5d orbitals compared to Mo4d states yields high carrier mobilities even within highly distorted, defect-rich structures, providing a wider electronic tuning window to effectively decouple the Seebeck coefficient from electrical conductivity. Finally, when synthesized in thin-film form, the reduction of ${WO}_{3}$ does not merely accumulate isolated point defects; instead, it triggers a periodic structural reorganization via crystallographic shear planes, giving rise to the sub-stoichiometric Magnéli phases. This unique phenomenon allows for the independent manipulation of electron and phonon transport paths. Consequently, ${WO}_{x}$ thin films serve as an ideal model system to isolate and systematically investigate the precise physical mechanisms that govern thermoelectric efficiency in oxide-based nanostructures.

To achieve the required defect architecture, pulsed laser deposition (PLD) is selected as a highly effective synthesis approach among the wide variety of available physical and chemical vapor deposition methods. The central advantage of PLD stems from its intrinsically far-from-equilibrium thermodynamic nature. The high kinetic energy of the ablated species within the laser-induced plasma plume enables the mobilization and subsequent “freezing” of metastable structural configurations and precise oxygen-deficient states that cannot be accessed via conventional equilibrium techniques. By fine-tuning the background oxygen partial pressure ($P_{O_{2}}$) and laser fluence, PLD provides a good and versatile non-equilibrium control over oxygen stoichiometry. This accurate thermodynamic manipulation strongly influences the kinetics of oxygen vacancy nucleation, forcing the self-assembly and ordering of these point defects into extended crystallographic shear planes. This controlled structural collapse ultimately drives the predictable formation of sub-stoichiometric tungsten Magnéli phases, unlocking defect-driven thermoelectric decoupling via the ’phonon-glass electron-crystal’ (PGEC) paradigm.

## 2. PLD-Grown Metal Oxides Thin Films and Their Thermoelectric Properties

The transport properties and localized structural alterations are intrinsically linked to the chemical and physical characteristics of the metal oxides (mainly as a thin film), which are deeply governed by the specific synthesis techniques employed during material processing. The tailoring of the physico-chemical properties, such as the density of crystallographic shear planes and oxygen vacancy networks, is fundamentally dictated by the thermodynamic and kinetic conditions of the synthesis process.

Building upon the comprehensive evaluation of state-of-the-art thermoelectric compositions and the underlying transport decoupling mechanisms in transition metal oxides detailed in the Appendix A and Appendix B, this section narrows the focus to metal oxide thin-film synthesized via PLD. After establishing a broad overview of PLD oxide films, we narrow our scope to tungsten oxide thin films, exploring how its substoichiometric phases can be precisely modulated to optimize thermoelectric performance. This choice moves from the knowledge that, while bottom-up wet-chemical routes offer precise atomic assembly and top-down techniques provide rapid macroscopic scaling, they both operate at or near thermodynamic equilibrium, limiting their ability to kinetically trap highly non-equilibrium defect states. To transcend these thermodynamic boundaries, advanced gas-phase processing, most notably pulsed laser deposition (PLD) under precisely controlled oxygen partial pressures or high vacuum, has emerged as a highly effective approach [7,8,9]. Furthermore, PLD operates under highly non-equilibrium kinetic conditions. The high-energy species within the laser-induced plasma plume allow for the trapping of metastable defect states and oxygen vacancy concentrations that transcend conventional thermodynamic solubility limits [10,11,12]. By exploiting epitaxial growth on lattice-matched single-crystal substrates, PLD enables the precise engineering of interfacial lattice strain. This strain fundamentally distorts the metal–oxygen orbital overlap, altering the electronic band symmetry to enhance the effective mass and systematically boost the Seebeck coefficient. In this review, pulsed laser deposition (PLD) is not considered as an industrial manufacturing method, but rather as a powerful research tool for exploring the physical limits of matter and understanding the decoupling mechanisms of transport parameters.

**Figure 1:**
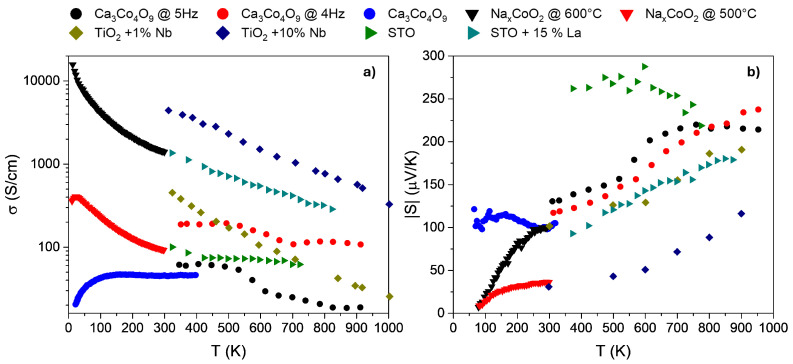
(**a**) Electrical conductivity and (**b**) Seebeck coefficient as a function of the temperature for several oxide materials synthesized by pulsed laser deposition. Data from Refs. [13,14,15,16,17,18].

Figure 1 shows the temperature dependence of electrical conductivity and Seebeck coefficient for representative layered oxides synthesized via PLD [13,14,15,16,17,18]. The misfit cobaltite $Ca_{3}Co_{4}O_{9}$ deposited on sapphire substrates via PLD represents one of the most promising thermoelectric materials for high-temperature energy conversion. Eng et al. investigated the thermoelectric properties of these thin films up to 130 °C under varying deposition conditions, including laser fluence (1.4–1.9 J/cm^2^), oxygen partial pressure (0.1–0.3 mbar), and substrate temperature (550–650 °C); they achieved minimum electrical resistivity at 600 °C and 1.7 J/cm^2^, independent of the oxygen deposition pressure [14]. Similarly, Sun et al. evaluated the transport behavior of the same system up to 700 °C, focusing on the influence of laser repetition rates. Working at a slower deposition rate (3–4 Hz), they reported optimized outcomes featuring a low electrical resistivity of 9.4 mΩ·cm and a remarkably high Seebeck coefficient of 240 μV/K at approximately 700 °C [13]. This performance highlighted how minimizing the deposition rate during PLD suppresses macroscopic droplet formation and promotes a highly ordered, well-crystallized layered structure, which drastically reduces grain-boundary scattering for charge carriers. Layered cobaltate ${\mathrm{Na}}_{x}{\mathrm{CoO}}_{2}$, depending on sodium concentration, possesses an unusually high thermoelectric power with low mobility, low resistivity, and high carrier density [19]. Venimadhav et al. studied the resistivity and Seebeck behaviors as a function of temperature for $Na_{x}CoO_{2}$ films grown by PLD at 500 °C and 600 °C [15].

Figure 1 shows that the highest conductivity and Seebeck values have been obtained for ${\mathrm{Na}}_{x}{\mathrm{CoO}}_{2}$ films grown at 600 °C. This optimization is closely linked to the precise stabilization of the sodium stoichiometry and volatile phase fractions within the PLD kinetic window, preventing the typical sodium-loss induced degradation observed in conventional bulk sintering. Beyond cobaltites, other PLD-prepared metal oxides heavily investigated for their effective thermoelectric performance include *La*- or *Nb*-doped strontium titanate ($SrTiO_{3}$) and anatase ($TiO_{2}$). Kurita et al. compared the thermoelectric properties of $TiO_{2}$ and $SrTiO_{3}$ under heavy Nb-doping conditions [16]. They revealed that while heavily *Nb*-doped anatase $TiO_{2}$ films exhibit a lower carrier effective mass ($m^{*}\approx 1m_{0}$), the degenerated *Ti*-$3d\;t_{2g}$ orbitals in the $TiO_{6}$ octahedral field of $SrTiO_{3}$ provide a significantly larger effective mass ($m^{*}\approx 10m_{0}$), allowing ${\mathrm{SrTiO}}_{3}$ to yield a superior thermoelectric power factor of $1.5\times 10^{-3}\;W\;m^{-1}\;K^{-2}$ at 900K. Concurrently, Ravichandran et al. conducted transport measurements on epitaxial $SrTiO_{3}$ thin films co-doped with La and oxygen vacancies over a temperature range of 300 to 900K [17]. By leveraging PLD to introduce a high concentration of oxygen vacancies alongside chemical doping, they successfully engineered a double-doped matrix that optimized carrier-transport dynamics, culminating in a remarkable dimensionless figure of merit ZT≈0.28 at 873K. Finally, a seminal study by Ohta et al. demonstrated that confining a high-density two-dimensional electron gas (2DEG) within a single-unit-cell layer thickness in $SrTiO_{3}$ generates an exceptionally high Seebeck coefficient (about 850 μV/K) while maintaining high electrical conductivity (about 1400S/cm) [18]. This enhancement originates from the localized low-dimensional density of states within the quantum well, showcasing PLD as an effective tool for atomic-scale structural tailoring to decouple intertwined thermoelectric transport parameters. This capacity of PLD to operate far from equilibrium and template atomic arrangements sets the stage for a comprehensive exploration of the sub-stoichiometric tungsten oxide ($WO_{x}$) system, where laser–matter interaction is exploited to trigger the self-assembly of tungsten Magnéli phases.

## 3. Structural and Electronic Evolution of Tungsten Magnéli Phases

**Figure 2:**
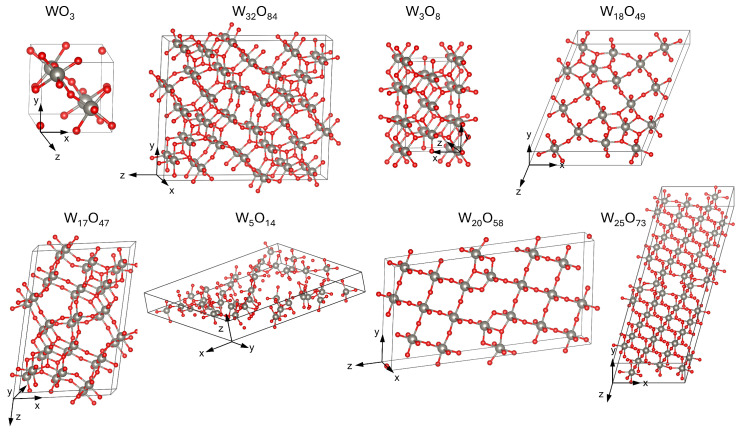
Unit cell models of tungsten oxide Magnéli phases. Color coding: tungsten atoms are shown as large gray spheres, and oxygen atoms as small red spheres.

A paramount manifestation of deterministic oxygen vacancy engineering can be observed in tungsten trioxide (${WO}_{3}$) and its sub-stoichiometric derivatives [20]. While pristine, stoichiometric ${WO}_{3}$ crystallizes as a wide-bandgap semiconductor characterized by an extremely low electrical conductivity ($\sigma \approx 2\;S\;{\mathrm{cm}}^{-1}$), the strategic extraction of oxygen atoms radically transforms its entire transport landscape into a series of non-stoichiometric phases designated as ${WO}_{3-x}$ (0<x<1). For slight oxygen deficiencies (x≤0.2), these sub-stoichiometric architectures, collectively known as Magnéli phases, typically organize into ordered crystallographic shear planes described by the homologous series formulas $W_{n}O_{3n-1}$ and $W_{n}O_{3n-2}$. Figure 2 shows the crystal lattice representations of these tungsten oxide Magnéli phases, where the large gray spheres denote tungsten atoms and the small dark spheres represent oxygen atoms. Experimentally, a wide variety of distinct ${WO}_{x}$ structures (2.625≤x≤2.92) have been identified, including $W_{32}O_{84}$, $W_{3}O_{8}$, $W_{18}O_{49},W_{17}O_{47},W_{5}O_{14},W_{20}O_{58},\mathrm{and}\;W_{25}O_{73}.$ Regarding their crystal symmetry, an orthorhombic structure is observed for $W_{32}O_{84}$ [21] and $W_{3}O_{8}$ [22], a tetragonal framework characterizes $W_{5}O_{14}$, while a monoclinic lattice uniquely distinguishes $W_{18}O_{49}$ [23], $W_{17}O_{47}$ [24], $W_{20}O_{58}$ [25], and $W_{25}O_{73}$ [26]. Conversely, $W_{5}O_{14}$ crystallizes in a tetragonal system [27]. A defining structural feature of these phases is the presence of $WO_{7}$ pentagonal bipyramids, which are encapsulated by five edge-sharing W−O octahedra [28].

Experimental data indicates that bulk $W_{18}O_{49}$ exhibits a metallic-like temperature dependence of electrical resistivity, with a reported charge carrier concentration of $1.87\times 10^{22}\;{\mathrm{cm}}^{-3}$. Similarly, metallic behavior has been identified in $W_{5}O_{14}$ nanowires using both photoelectron spectroscopy and transport measurements [23,29].

**Figure 3:**
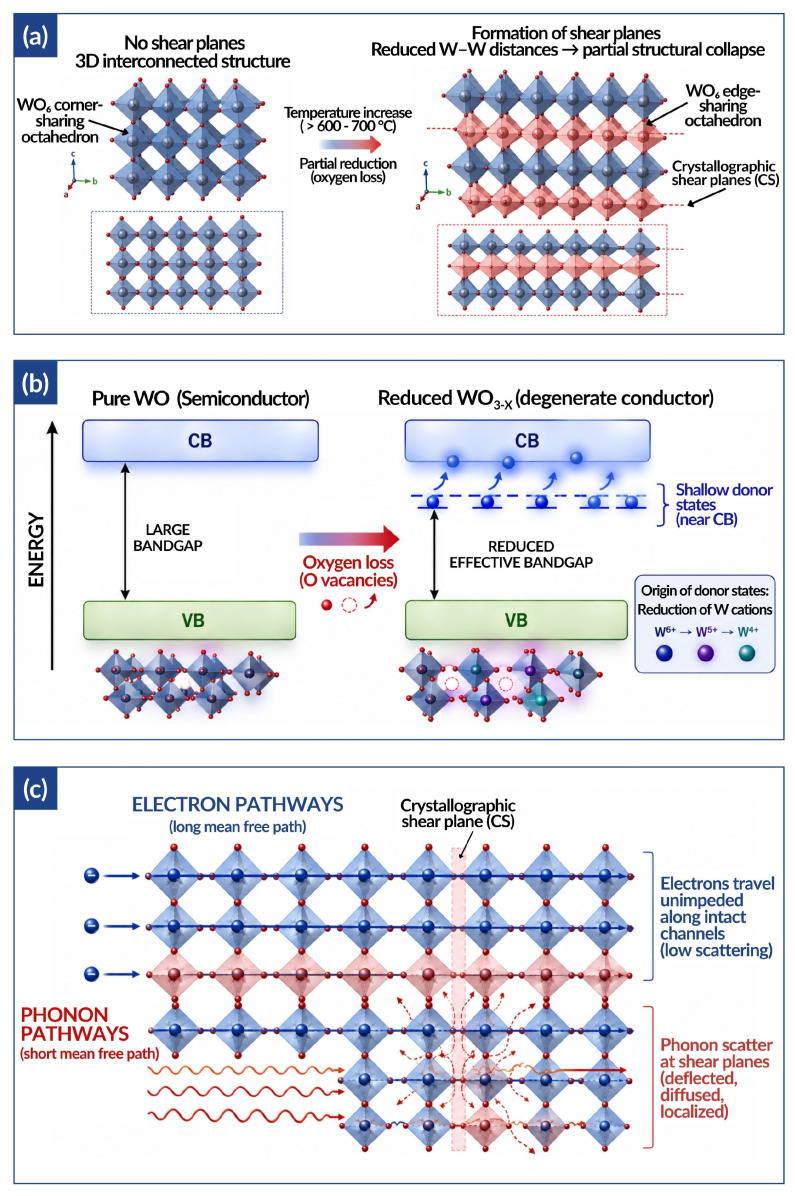
Schematic representation of transport engineering in tungsten-based Magnéli phases: (**a**) Structural transition from corner-sharing $WO_{6}$ octahedra in stoichiometric $WO_{3}$ to edge-sharing configurations along crystallographic shear (CS) planes due to oxygen vacancy alignment. (**b**) Schematic electronic band structure modification showing the emergence of shallow donor states ($W^{5+}/W^{4+}$) near the conduction band minimum. (**c**) Illustration of the phonon-glass electron-crystal paradigm, where CS planes selectively scatter acoustic phonons while preserving coherent electronic pathways.

For example, when oxygen vacancies are systematically introduced into the perovskite-like $WO_{3}$ matrix within a light reduction regime (0<x≤0.2), the crystal lattice undergoes a periodic, collective collapse along specific crystallographic planes to minimize intense local strain [30,31,32,33]. This structural rearrangement effectively eliminates a complete plane of oxygen vacancies, replacing the original corner-sharing framework with a localized, highly dense network of edge-sharing $WO_{6}$ octahedra known as crystallographic shear (CS) planes (Figure 3a). The resulting compounds conform to precise homologous series typically defined by the general formulas $W_{n}O_{3n-1}$ and $W_{n}O_{3n-2}$, which constitute the true tungsten Magnéli phases [31,34,35,36], already classified (Figure 2). This shear phenomenon drives advanced transport decoupling across two parallel physical vectors involving electronic injection and phononic transport barrier conversion. In the electronic vector, the formation of oxygen vacancies forces the reduction of adjacent multivalent tungsten cations from the $W^{6+}$ state into lower $W^{5+}$ or $W^{4+}$ valence states, creating shallow donor states pinned near the conduction band minimum (Figure 3b).

The result is a massive influx of free charge carriers that shifts the material from a poorly conducting semiconductor to a heavily doped, metallic-like n-type conductor, with σ reaching almost $1000\;S\;{\mathrm{cm}}^{-1}$ [36,37,38]. Concurrently, in the phononic vector, rather than allowing disordered, random point defects to scatter charge carriers destructively, the resulting periodic crystallographic shear planes act as specialized, coherent nanoscale boundaries. To place this defect-driven transport decoupling into a broader theoretical and empirical context, it is instructive to evaluate how sub-stoichiometric transitions alter both localized electronic environments and collective lattice dynamics. The transformation of point defects into highly ordered, extended planar structures fundamentally redefines the scattering landscape. Spatially, the edge-sharing $WO_{6}$ octahedra concentrated along these shear boundaries are believed to facilitate continuous d-orbital overlap pathways that promote efficient charge transport. At the same time, the long-range periodicity and mass-density modulation associated with crystallographic shear (CS) planes act as effective scattering centers that suppress the propagation of acoustic phonons. This phenomenon is consistent with strategies observed in other transition metal sub-oxides, such as Magnéli-phase titanium oxides ($TiO_{2-x}$), where ordered oxygen deficiency and shear-plane networks significantly reduce lattice thermal conductivity while preserving high electrical transport performance [39]. Comparable behavior has also been observed in tungsten oxide systems, where the formation of shear-plane-containing substoichiometric phases leads to reduced thermal conductivity while preserving efficient electrical transport [36,40]. Furthermore, the structural evolution from stoichiometric $WO_{3}$ to oxygen-deficient phases induces measurable changes in lattice vibrational properties. The reduction of $W^{6+}$ to mixed $W^{5+}/W^{4+}$ states introduces local symmetry breaking and lattice distortions, which are reflected in the broadening and shifting of characteristic W−O stretching and bending vibrational modes. These spectroscopic signatures are indicative of increased lattice disorder and enhanced phonon scattering, contributing to the suppression of heat-carrying phonon transport [41]. Consequently, the observed reduction of lattice thermal conductivity arises not from amorphization but from the deterministic formation of an engineered nanoscale boundary network that enforces a phonon-glass electron-crystal (PGEC) transport regime. Thus, a rigorous validation of this defect-driven transport decoupling requires a multi-scale correlation between structural and electronic properties. Spectroscopic techniques such as X-ray photoelectron spectroscopy (XPS) and X-ray absorption near-edge structure (XANES) confirm the progressive reduction of tungsten oxidation states induced by oxygen vacancies. This electronic evolution is consistent with transport measurements showing increased carrier densities and the transition from localized polaronic conduction to more delocalized, metallic-like transport regimes [37,42]. Concurrently, High-Resolution Transmission Electron Microscopy (HRTEM) directly reveals the periodic and coherent nanoscale arrangement of crystallographic shear planes characteristic of Magnéli phases. These structural features introduce localized strain fields and symmetry breaking, which are reflected in Raman spectroscopy through the broadening and frequency shifts of W−O vibrational modes. These features indicate enhanced phonon scattering and lattice disorder at multiple length scales. Crucially, this structural disruption directly correlates with macroscopic transport behavior, as experimentally demonstrated in shear-plane-rich tungsten oxides exhibiting suppressed lattice thermal conductivity approaching the amorphous limit [36]. The combined structural and spectroscopic evidence provides strong support for a unified physical picture in which crystallographic shear planes selectively scatter heat-carrying phonons while preserving efficient electronic transport pathways, thereby enabling defect-driven thermoelectric decoupling in tungsten Magnéli phases. The planar defect structures intensely scatter mid-to-high frequency heat-carrying acoustic phonons (Figure 3c). Consequently, sub-stoichiometric $WO_{3-x}$ Magnéli phases exhibit an exceptionally low lattice thermal conductivity ($\kappa_{L}\approx 1.2–1.6\;W\;m^{-1}K^{-1}$), approaching the theoretical amorphous limit of the oxide without severely degrading the extended electronic pathways [42]. Conversely, upon deeper reduction (x>0.2), the system undergoes a radical structural transition away from crystallographic shear, culminating in the distinct, monoclinic $W_{18}O_{49}$ phase [43,44]. Unlike the planar topologies of the Magnéli series, $W_{18}O_{49}$ accommodates its extreme oxygen deficiency through an intricate three-dimensional framework of edge-, corner-, and face-sharing $WO_{6}$ octahedra that form pentagonal columns and large intracrystalline. This atomic rearrangement acts as a strong source of carrier scattering, which severely curtails carrier mobility and drives a sharp increase in electrical resistivity. Simultaneously, while the extreme oxygen deficiency injects a high concentration of free electrons into the system, the pervasive structural disorder and potential energy filtering effects fail to elevate the Seebeck coefficient enough to counteract the resistivity penalty. Because the power factor PF is mathematically defined by the square of the Seebeck coefficient divided by electrical resistivity, this disproportionate surge in resistivity severely suppresses the material’s electronic efficiency. Ultimately, while this tunnel architecture maintains low lattice thermal conductivity by heavily damping acoustic phonon modes, its unique atomic configuration disrupts the coherent transport of charge carriers compared to the highly oriented Magnéli shear paths, thereby locking the material into a specific transport ceiling.

## 4. Morphological Classification and Dimensionality Effects on Transport Properties of ${\mathrm{WO}}_{x}$ Thin Films

When evaluating the electrical and thermoelectric performance of tungsten oxide systems, a rigorous distinction must be made regarding the structural form of the material, as synthesis pathways and geometric dimensionality fundamentally dictate the physical transport mechanisms across distinct operating temperature windows. In sintered bulk tungsten oxide ceramics, typically densified via Spark Plasma Sintering, charge and heat transport are governed by three-dimensional lattice connectivity and are strongly influenced by grain boundary scattering. Partial reduction induces the formation of extended crystallographic shear-plane networks and oxygen vacancies, driving the electrical conductivity toward heavily doped, metallic-like regimes. In these oxygen-deficient Magnéli phases, conductivities approaching metallic-like values (up to ∼$10^{2}$–$10^{3}$ S cm^−1^ depending on oxygen stoichiometry and phase composition) have been reported, reflecting the high carrier densities associated with $W^{5+}/W^{4+}$ states [36,45]. Within high-temperature regimes (300–1000 K), these bulk tungsten sub-oxides maintain structural stability and efficient transport, while the ordered shear boundaries suppress lattice thermal conductivity across the entire temperature range [36]. Conversely, tungsten oxide thin films are governed by nanoscale size effects and interfacial phenomena. Their transport properties are highly sensitive to structural evolution, particularly the transition from amorphous to crystalline $WO_{3}$ phases, which typically occurs upon thermal annealing in the range of ∼500–600 K, depending on deposition conditions and film structure [46,47]. As the film thickness approaches the mean free paths of charge carriers and phonons, surface and interface scattering significantly alter transport behavior relative to bulk systems. Furthermore, interfacial thermal resistance and nanoscale structural disorder strongly suppress heat transport. Experimental measurements of $WO_{3}$ thin films have demonstrated thermal conductivity values in the range of ∼1.28–1.63 W m^−1^K^−1^, depending on phase composition and interface effects [48]. These reduced values originate from enhanced phonon scattering due to structural disorder, grain boundaries, and phase heterogeneity, which limit phonon mean free paths. An entirely different transport landscape emerges in one-dimensional tungsten oxide nanostructures, such as nanowires and nanotubes. In these systems, the extremely high surface-to-volume ratio and reduced dimensionality introduce strong boundary scattering effects. Nanoscale confinement and defect-induced disorder significantly enhance phonon scattering mechanisms, leading to reduced lattice thermal conductivity compared to bulk counterparts, as generally observed in nanostructured oxide systems [48,49]. At the same time, the localized nature of charge carriers in these reduced-dimensional systems often leads to hopping-dominated transport, deviating from conventional band conduction and aligning with disordered transport models [37,42]. Consequently, direct comparison of thermoelectric parameters such as electrical conductivity, the Seebeck coefficient, and the figure of merit across bulk, thin-film, and nanostructured tungsten oxides must be approached with caution. Localized enhancements observed in thin films or nanoscale systems often arise from interfacial or confinement-driven effects and cannot be directly extrapolated to bulk thermoelectric performance under high-temperature operating conditions.

## 5. Tuning of Structural and Thermoelectric Properties of PLD Grown ${\mathrm{WO}}_{x}$ Thin Films

While alternative synthesis strategies such as magnetron sputtering, atomic layer deposition (ALD), chemical vapor deposition (CVD), sol–gel processing, and hydrothermal synthesis provide versatile routes for fabricating tungsten oxide materials, pulsed laser deposition (PLD) offers distinct advantages for probing structure–property correlations under controlled conditions. Wet chemical methods, including sol–gel and hydrothermal approaches, are particularly effective for large-scale synthesis of tungsten oxide nanostructures. However, these techniques often lead to variations in stoichiometry, residual hydroxyl species, and structural heterogeneity associated with hydration and incomplete crystallization, which can obscure the intrinsic transport response of oxygen-deficient tungsten oxides [50]. In contrast, vacuum-based deposition methods such as CVD and magnetron sputtering provide improved control over film uniformity and large-area scalability. Nevertheless, these techniques may face challenges in maintaining precise stoichiometry during film growth, particularly under conditions where oxygen vacancy formation is highly sensitive to process parameters [51]. Atomic layer deposition (ALD) offers exceptional thickness control and conformal coating capabilities at the atomic scale. However, its growth mechanism, typically based on self-limiting surface reactions and precursor chemistry, can restrict the range of accessible non-equilibrium oxidation states and limit the formation of highly reduced phases necessary for achieving metallic-like transport in tungsten sub-oxides [51]. Within this synthesis landscape, PLD emerges as a uniquely versatile method for investigating the interplay between defects and transport properties. The congruent transfer characteristic of laser ablation enables the faithful reproduction of complex target stoichiometry in the deposited film. Moreover, the high kinetic energy of ablated species promotes non-equilibrium growth conditions, which can be systematically tuned through parameters such as oxygen partial pressure and laser fluence, allowing controlled generation of oxygen vacancies and defect structures [51]. This capability is particularly relevant for tungsten Magnéli phases, where the controlled formation of oxygen-deficient networks and crystallographic shear planes has been shown to govern the electrical and thermal transport properties over a broad temperature range (300–1000 K) [36]. Furthermore, PLD is especially well suited for engineering nanoscale features such as grain boundaries, epitaxial strain, and interfacial coupling in tungsten oxide thin films. These factors enable the decoupling of electronic and phononic transport pathways, facilitating systematic investigations of defect-mediated transport mechanisms. In particular, the role of localized charge carriers and defect-induced transport, including polaronic conduction mechanisms, can be directly correlated with structural features, as described in disordered oxide systems [37,42]. Ultimately, PLD provides a controlled experimental platform for exploring non-equilibrium defect configurations that are difficult to access via equilibrium synthesis routes. The landscape of oxide thermoelectrics has dramatically evolved over the last five years, with a precise shift from macro-scale stoichiometry control to sub-nanometer defect architecture manipulation in ${WO}_{3-x}$ systems [2,52]. The recent literature emphasizes the use of pulsed laser deposition (PLD) not merely as a deposition tool, but as a dynamic synthesis route to stabilize highly non-equilibrium structures [50,53]. Breakthrough studies have demonstrated that the non-equilibrium kinetic profile of the PLD plasma plume allows for the precise injection of ordered oxygen vacancies ($O_{v}$), driving a controlled structural collapse into precise tungsten Magnéli phases, such as $W_{18}O_{49}$ and $W_{25}O_{73}$ [41,52,54]. Advanced depth-resolved spectroscopic and ellipsoidal modeling tracks how these ordered defect clusters create unique sub-bandgap electronic states near the Fermi level [55]. This specific quantum confinement preserves high carrier mobilities via the extended spatial overlap of *W* 5d electron orbitals even when the structural bond angles fluctuate [52], thereby maximizing the Seebeck coefficient through sharp changes in the density of states. Concurrently, recent computational and experimental benchmarks from 2024–2026 have clarified acoustic phonon transport across crystallographic shear planes [41]. These localized planar networks induce giant acoustic impedance mismatches, selectively suppressing the lattice thermal conductivity ($\kappa_{L}$) toward the amorphous limit without restricting the metallic-like electronic pathway [2,52]. Furthermore, recent studies on oxygen-deficient tungsten oxides and related Magnéli phases have shown that crystallographic shear planes, arising from ordered oxygen vacancies, enhance high-temperature stability by suppressing structural relaxation and thermal conductivity, while contributing to defect stabilization over a wide temperature range (up to about 1000–1100 K) [36,40,56]. The controlled induction of oxygen vacancies during the PLD process transforms the intrinsically insulating, stoichiometric $WO_{3}$ into a highly degenerate n-type semiconductor or a semi-metal [57]. At the microscopic level, the laser-driven extraction of oxygen atoms releases free electrons into the 5d conduction band, forcing an increase in electrical conductivity across several orders of magnitude, from $10^{-6}\;S\;{\mathrm{cm}}^{-1}$ up to peaks of $10^{3}\;S\;{\mathrm{cm}}^{-1}$ [58,59] (see Table 1).

The available PLD literature highlights oxygen partial pressure as the key deposition parameter governing the structural, electronic, and thermoelectric properties of $WO_{x}$ thin films. In general, decreasing oxygen partial pressure promotes the formation of oxygen-deficient $WO_{x}$ phases, resulting in a dramatic increase in electrical conductivity, whereas increasing oxygen pressure and/or substrate temperature favors the formation of increasingly stoichiometric and crystalline $WO_{3}$ phases characterized by larger optical band gaps, lower electrical conductivity, and higher crystallinity. An overview on the physico-chemical properties influencing the transport/thermoelectrical response is provided in Table 1. It is worth to note that conductivity values spanning more than seven orders of magnitude are reported across the investigated pressure range, evidencing the exceptional sensitivity of PLD-grown $WO_{x}$ to oxygen-vacancy concentration. Notably, Kim et al. provide the only PLD study reporting electrical conductivity, Seebeck coefficient and thermal conductivity on the same $WO_{x}$ thin-film system, enabling a complete thermoelectric assessment. Based on the reported data, power factors of the order of 10^−5^–10^−4^ W m^−1^ K^−2^and ZT values in the 10^−3^–10^−2^range can be estimated [58,59].

On the overall, electrical transport in PLD-grown $WO_{x}$ thin films is strongly governed by oxygen-vacancy concentration and film stoichiometry. Highly reduced $WO_{3-x}$ phases exhibit markedly enhanced electrical conductivity due to the increased density of donor-like oxygen vacancies, whereas nearly stoichiometric $WO_{3}$ and amorphous $WO_{x}$ films are characterized by localized charge transport and significantly lower carrier concentrations. The relationship between the crystallographic shear planes and the resulting thermoelectric properties in tungsten Magnéli phases (${WO}_{3-x}$) is governed by a delicate balance of quantum mechanical transport, structural scattering, and lattice dynamics. In stoichiometric ${WO}_{3}$, the electrical resistivity is high because the valence band consists of fully occupied oxygen 2p orbitals and the conduction band remains empty. As oxygen atoms are systematically removed, the structural collapse into edge-sharing octahedra frees up electrons that previously participated in covalent bonds, as previously reported. The localized charge distribution can be described by the small polaron hopping model at higher temperatures or intermediate reduction levels, where the electrical resistivity follows an Arrhenius-type behavior given by

(1) $$\rho \left(T\right)=\rho_{0}\exp\left(E_{a}/k_{B}T\right),$$

where $E_{a}$ is the activation energy for hopping and $k_{B}$ is the Boltzmann constant [67]. However, as the system transitions into the highly ordered Magnéli phases such as $W_{20}O_{58}$ or $W_{18}O_{49}$, the extreme density of crystallographic shear planes yields a completely delocalized electron gas within the 5d band of the tungsten ions. In this metallic regime, the resistivity transitions to a traditional linear or power-law dependence with temperature, where

(2) $$\rho \left(T\right)=\rho_{0}+AT^{n},$$

as electron-phonon and electron-defect scattering processes become dominant. The evolution of the Seebeck coefficient (*S*) across these distinct phases is mathematically coupled to this electronic transformation through the Mott formula, which defines the thermoelectric power for a degenerate electron system [68]. This relationship states that

(3) $$S=\frac{\pi^{2}k_{B}^{2}T}{3e}{\left[\frac{d\ln\sigma (E)}{dE}\right]}_{E=E_{F}},$$

where σ(E) represents the energy-dependent electrical conductivity and $E_{F}$ is the Fermi level. By breaking down the conductivity into the product of the electronic density of states N(E) and the carrier mobility μ(*E*), the equation simplifies to show that the Seebeck coefficient is directly proportional to the energy derivative of the density of states at the Fermi level, written as

(4) $$S\propto {\left[\frac{1}{N(E)}\frac{dN(E)}{dE}+\frac{1}{\mu (E)}\frac{d\mu (E)}{dE}\right]}_{E=E_{F}}.$$

In the slightly reduced phases like ${WO}_{2.96}$ or $W_{25}O_{73}$, the Fermi level sits near the very bottom of the conduction band where the slope dN(E)/dE is exceptionally steep, yielding a large, negative Seebeck coefficient indicative of *n*-type conduction. As the reduction progresses toward $W_{18}O_{49}$, the massive influx of free carriers fills the 5d band, shifting $E_{F}$ deep into the metallic zone. This drastic increase in carrier concentration causes $N(E_{F})$ to swell significantly while flattening the derivative dN(E)/dE, causing a drastic reduction in the absolute magnitude of the Seebeck coefficient. The optimization of the thermoelectric power factor ($PF=S^{2}/\rho$) relies on finding the exact stoichiometry where the product of these competing variables peaks. In the free electron approximation, the carrier concentration *n* dictates that the resistivity scales inversely with carrier density as $\rho =(ne\mu {)}^{-1}$, while the Seebeck coefficient scales as $S\propto n^{-2/3}$. When evaluating the power factor as a function of the chemical potential, the mathematical expression becomes $PF\left(n\right)\propto n^{1/3}\mu$, illustrating that a maximum value is achieved when the carrier density reaches an intermediate regime between $10^{19}$ and $10^{21}\;{\mathrm{cm}}^{-3}$ [69]. In the tungsten Magnéli phases, this critical electronic window corresponds to the intermediate structures like $W_{20}O_{58}$ and $W_{5}O_{14}$. In these specific structural arrangements, the complex edge-sharing and face-sharing configurations of the ${WO}_{6}$ octahedra induce sharp van Hove singularities in the electronic density of states. Computational density functional theory (DFT) models utilize advanced functionals, such as the screened hybrid functional HSE06 or DFT + U methods to account for strongly correlated 5d electrons, confirming that these localized structural distortions generate sudden, step-like increases in N(E). These quantum mechanical features preserve a high dN(E)/dE value to maintain a respectable Seebeck coefficient, while the continuous chains of corner-sharing octahedra run parallel to the shear planes to serve as high-mobility channels that minimize electrical resistivity. Maximizing the overall thermoelectric efficiency relies heavily on suppressing the lattice thermal conductivity ($\kappa_{L}$), a task where the intrinsic architecture of Magnéli phases excels. The total thermal conductivity is described as $\kappa =\kappa_{e}+\kappa_{L}$, where the electronic contribution $\kappa_{e}$ scales proportionally with electrical conductivity via the Wiedemann–Franz law, $\kappa_{e}=L\sigma T$, where *L* is the Lorenz number. To decouple the transport parameters, the lattice thermal conductivity must be selectively minimized according to the Callaway model, which expresses $\kappa_{L}$ as an integral over the phonon frequency spectrum weighted by a total relaxation time $\tau_{C}\left(\omega \right)$. This total relaxation rate is governed by Matthiessen’s rule, combining various scattering mechanisms such that

(5) $$\tau_{C}^{-1}=\tau_{U}^{-1}+\tau_{B}^{-1}+\tau_{PD}^{-1}+\tau_{CSP}^{-1},$$

where the individual terms represent Umklapp scattering, boundary scattering, point-defect scattering from oxygen vacancies, and crystallographic shear plane scattering respectively. The unique atomic arrangement of the shear planes introduces a highly periodic nanostructuring that matches the mean free path of mid-to-low frequency acoustic phonons, which are responsible for the bulk of heat transport. High-temperature experimental data collected via Spark Plasma Sintering (SPS) processing confirms that phases such as ${WO}_{2.90}$ exhibit ultra-low lattice thermal conductivities (reaching values between 1.5 and 2.5 $W\;m^{-1}K^{-1}$). These internal interfaces act as heavy boundary scatterers that disrupt coherent phonon propagation without causing a corresponding devastation to electron mobility along the intact octahedral paths, driving the lattice thermal conductivity toward the theoretical amorphous limit and generating competitive thermoelectric figures of merit (ZT) at elevated temperatures [40,70,71,72].

**Figure 4:**
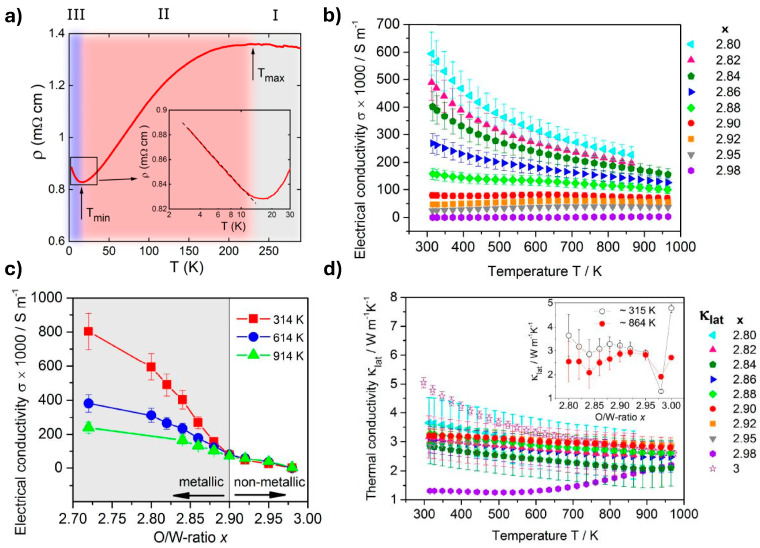
(**a**) Resistivity as a function of temperature for the ${WO}_{2.9}$ sample. Inset: Expanded view of the low-temperature region shown with a logarithmic horizontal scale. Dashed line is a guide for the eye. (**b**) Electrical conductivity σ(T) of the ${WO}_{x}$ samples (2.80≤x≤2.98); (**c**) The temperature dependence of σ(T) clearly changes from non-metallic to metallic at x ≃ 2.90. (**d**) Lattice contribution $\kappa_{lat}=\kappa_{tot}-\kappa_{el}$ (with $\kappa_{el}$ from the Wiedemann–Franz law) of the $WO_{x}$ samples (2.80≤x≤3). The inset shows the dependency $\kappa_{lat}\left(x\right)$ for 315 K and 864 K. Panel (**a**) from Ref. [73] and panels (**b**–**d**) form Ref. [40] under the terms of the CC BY 4.0 license.

This comprehensive theoretical framework directly correlates with the experimental observations reported by Shengelaya et al. [73] on dense ${WO}_{2.9}$ bulk samples. Their transport analysis reveals an unconventional transition from a metallic-like regime (ρ proportional to T*^n^* as described by Equation (2)) at lower temperatures (T < 16 K, regime III) to a resistivity saturation (16 K < T < 203 K, regime II) and a negative temperature coefficient near and above room temperature (T > 230 K, regime I) (Figure 4a). This electronic crossover is governed by the Mott–Ioffe–Regel limit, where the carrier mean free path approaches the interatomic distance. Such behavior provides a solid physical justification for the application of the Arrhenius-type small polaron hopping formalism (Equation (1)) as an effective phenomenological tool to describe the localized charge transport in intermediate reduction regimes at fixed thermoelectric operation temperatures. Furthermore, magnetic and electronic structure data reported by Shengelaya et al. confirm that the *W* 5d states immediately surrounding the crystallographic shear planes dominate the density of states at the Fermi level. This structural localization generates sharp energy gradients (dN(E)/dE) that mathematically validate the Mott formulation in Equations (3) and (4), enabling the optimization of the Seebeck coefficient and the power factor within the ${WO}_{2.9}$ Magnéli phase.

This structure–property relationship is strongly corroborated by the work of Kaiser et al. [40], who systematically investigated ${WO}_{x}$ compounds (2.50≤x≤3) processed via Spark Plasma Sintering. As shown in Figure 4b,c, the electrical conductivity strongly correlates with the O/W-ratio x. For x≥2.90, σ(T) continuously decreases without net dependence on temperature; furthermore, the limit between metallic and nonmetallic behaviour is just observed for the O/W-ratio of 2.90. Their high-temperature data explicitly show that lattice thermal conductivity is minimized and continuously tunable near 700 K within the sub-stoichiometric ${WO}_{2.84}$ phase (Figure 4d). This reduction occurs via CS planes (corner-sharing coordination is replaced by edge-sharing sub-structures) that act as internal physical boundaries suppressing heat transport but preserving electrical conductivity through metallic W−W bonds. These new structural reorganization introduces spatial defects which act as native phonon-scattering centers, satisfying the mathematical requirements of the Callaway model (Equation (5)) which can be used to quantify the reduction in lattice thermal conductivity as a function of the spatial inter-planar distance between these shear boundaries.

**Figure 5:**
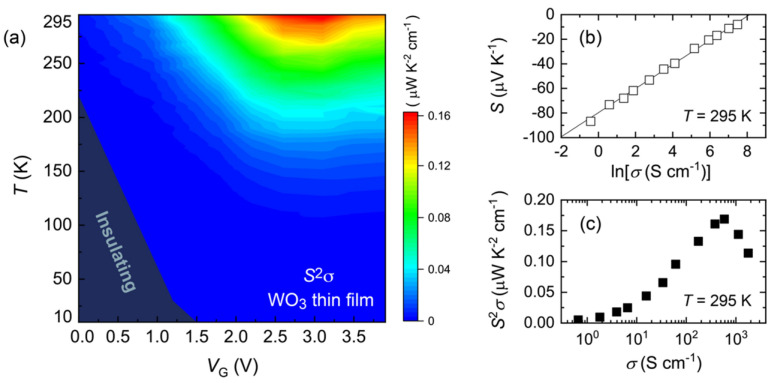
Optimization of thermoelectric power factor in $WO_{3}$ thin film. (**a**) Mapping of thermoelectric power factor $S^{2}\sigma$ against temperature T and gate voltage VG. (**b**) Evolution of S against electrical conductivity σ showing how S changes linearly with ln(σ). (**c**) Optimization of $S^{2}\sigma$ of $WO_{3}$ at room temperature. Figure reprinted from Ref. [71] under the terms of the CC BY 4.0 license.

On the electronic transport side, the validity of Equations (3) and (4) is further supported by the experimental gating studies on ${WO}_{3}$ thin films [71]. By precisely controlling the carrier concentration through electrochemical gating, Shimizu et al. continuously shifted the Fermi level and tracked the evolution from insulating to metallic transport. The observed maximum in power factor occurred in the highly degenerate regime, where the energy dependence of the electronic density of states near the Fermi energy, as described by the Mott relation, provides an optimal compromise between high electrical conductivity and retention of a measurable Seebeck coefficient. In particular, Shimizu et al. [71] demonstrate, by a machine learning approach, that the maximum PF occurs at a gate voltage ($V_{G}$) of ∼3.1 V, where the material enters a degenerate metallic state (Figure 5a). While PF increases with temperature, PF reaches an optimal point at $V_{G}\approx 3.1\;V$ before performance degrades from over-doping, with the Seebeck coefficient showing a linear S∝−ln(σ) relationship (Figure 5b). The study confirms maximum room-temperature PF ($\approx 0.16\;\mu W\;K^{-2}\;{\mathrm{cm}}^{-1}$) occurs at a conductivity of ∼585 $S\;{\mathrm{cm}}^{-1}$ (Figure 5c), identifying the optimal balance in a highly degenerate metallic phase [71]. Collectively, these independent experimental studies on both bulk and thin-film tungsten sub-oxides provide concrete empirical evidence that links our theoretical transport equations directly to the structural dynamics of the synthesized samples at fixed operating temperatures.

In this context, pulsed laser deposition significantly enhances the thermoelectric efficiency of metal oxide thin films by fundamentally decoupling the interconnected transport variables of the figure of merit through distinct microscopic and quantum mechanisms [74]. In bulk materials, increasing electrical conductivity typically compromises the Seebeck coefficient and increases thermal conductivity, but the high energy and precise atomic control of this PLD deposition technique allow researchers to overcome these classical limitations. By restricting film growth to sub-nanometer scales, the technique induces quantum confinement effects that transition the electronic density of states from a continuous three-dimensional profile to a sharp, step-like two-dimensional structure. This sharp energy distribution drives a massive spike in the Seebeck coefficient because the thermopower is directly proportional to the energy derivative of this density of states near the Fermi level, allowing for high voltage generation without sacrificing electrical performance. Simultaneously, the process exploits the massive disparity between the mean free paths of electrons and phonons to realize a phonon-glass electron-crystal paradigm [75,76,77]. Because heat-carrying phonons possess a significantly longer mean free path than charge-transporting electrons in metal oxides, the precise introduction of artificial interfaces, grain boundaries, and superlattice periodicities scatters these lattice vibrations and heavily depresses the lattice thermal conductivity [18]. Electrons, possessing a much shorter mean free path, pass through these interfaces relatively unimpeded, thereby maintaining robust electrical conductivity. This transport is further optimized through the unique high-energy plume physics inherent to the laser ablation process, which enables precise control over point defects and oxygen vacancies. Practically, by tuning the background oxygen pressure during growth, native oxygen vacancies are introduced in the tungsten oxide matrix. These oxygen vacancies inject high carrier concentrations directly into the lattice without destabilizing the host structure. These nanoscale interfaces can also be engineered to induce an energy filtering effect, where structural potential barriers selectively filter low-energy electrons while allowing highly energetic hot electrons to pass, which increases the average energy transported per carrier and raises the overall Seebeck coefficient [78,79]. Finally, the ability to achieve high-quality epitaxial growth creates structural strains between the film and the underlying substrate, distorting the oxide’s unit cell, altering its band structure, and lowering the effective mass of the carriers to substantially increase electron mobility.

We remark that the highly directional nature of the laser-induced plasma plume naturally creates severe spatial variations across the substrate area, resulting in pronounced thickness and compositional stoichiometry gradients. Because the electrical transition from a semiconductor to a highly degenerate metal is extremely sensitive to minute fluctuations in oxygen vacancy content, these gradients can cause a single thin film to drift unpredictably between target Magnéli phases and poorly conducting regions. Mitigating this spatial anisotropy requires the strict implementation of plume-shaping geometries, such as optimized shadow masks or customized gas nozzle configurations, working in tandem with continuous substrate rotation to guarantee a spatially uniform flux of ablation species over large-area deposits. Beyond the spatial control of film morphology, a major obstacle hindering the integration of these materials into practical high-temperature energy harvesters is their thermochemical vulnerability under ambient conditions. Although optimized tungsten Magnéli phases demonstrate remarkably high thermoelectric efficiency and exceptional power factors around 1100 K, their sub-stoichiometric lattice remains highly unstable when exposed to an oxygen-rich atmosphere at elevated operational temperatures. The open structural channels and active internal surfaces characterising the crystallographic shear planes lower the energy barrier for gas absorption, forcing a rapid, spontaneous re-oxidation process that reverts the highly conductive metallic sub-oxides back into the intrinsically insulating stoichiometric ${WO}_{3}$ phase. Preventing this degradation requires the design of advanced oxygen-impermeable encapsulation layers, such as highly dense aluminum oxide (${Al}_{2}O_{3}$) deposited via atomic layer deposition or specialized, thermally matched amorphous silicate coatings. These protection barriers act as robust, microstructurally sound diffusion seals that isolate the core ${WO}_{3-x}$ film from environmental oxygen ingress, successfully preserving the underlying edge-sharing octahedra chains and maintaining stable, degradation-free performance during long-term thermal cycling [58,80,81].

## 6. Multifunctional Application Prospects of PLD-Grown Tungsten Magnéli Phases

The precise structural tailoring and defect engineering required for thermoelectric decoupling, such as the modulation of oxygen vacancy density and the ordering of crystallographic shear planes, inherently affect the electronic and optical band structures of tungsten Magnéli phases. Consequently, the same physical mechanisms that decouple electrical conductivity from thermal transport also open up versatile pathways for multifunctional applications, bridging fundamental transport physics with cross-disciplinary technologies. While the primary focus of this review centers on defect-driven thermoelectric decoupling, the unique physical chemistry of pulsed laser deposition (PLD)-grown oxygen-deficient ${WO}_{x}$ and tungsten Magnéli phases extends across multiple advanced technological domains. The far-from-equilibrium growth conditions enable precise control over ordered oxygen vacancies ($O_{v}$) and crystallographic shear planes, resulting in highly tunable electronic structures, high carrier densities, and enhanced functional properties across energy, catalytic, and electronic applications [51,82]. Beyond thermoelectrics, sub-stoichiometric ${WO}_{3-x}$ thin films are emerging as promising electrode materials for energy storage. In particular, tungsten oxide thin-film electrodes exhibit strong potential in flexible supercapacitors due to their high theoretical capacitance, structural versatility, and favorable charge-storage mechanisms [83]. The presence of crystallographic shear planes, characteristic of Magnéli-type structures, facilitates electronic and ionic transport by providing extended defect pathways and reducing transport barriers [36]. Moreover, nanoscale thickness control in ALD- and PLD-derived systems critically governs rate capability and pseudocapacitive behavior [84].

Defect-engineered tungsten suboxides also play a key role in electrocatalysis. Non-stoichiometric transition metal oxides with high densities of oxygen vacancies exhibit enhanced catalytic performance, as defect-rich surfaces can lower adsorption energy barriers for key intermediates in reactions such as the hydrogen evolution reaction (HER) and oxygen evolution reaction (OER) [85]. The tunability of oxidation states in tungsten Magnéli phases further enables optimization of active catalytic sites. In parallel, oxygen-deficient ${WO}_{x}$ thin films are widely investigated for optoelectronic and electrochromic applications. Their high carrier concentration and tunable band structure make them attractive materials for smart windows, where fast and reversible intervalence charge transfer ($W^{5+}↔W^{6+}$) enables high coloration efficiency and long-term cycling stability [86].

Beyond energy and optoelectronic applications, the high density of surface defects and oxygen vacancies also makes tungsten suboxides excellent candidates for gas sensing technologies. The enhanced adsorption–desorption kinetics at vacancy sites significantly improves sensitivity and selectivity toward gases such as $H_{2}$, CO, and volatile organic compounds [87]. Furthermore, the defect-tailored electronic structure of ${WO}_{3-x}$ enables efficient light absorption and charge separation, making these materials suitable for photocatalytic and photoelectrochemical applications [88,89,90]. Oxygen vacancies introduce sub-bandgap states that extend optical absorption into the visible region, thereby enhancing solar-driven processes such as water splitting [91]. At the device level, the coupled ionic–electronic transport enabled by ordered oxygen vacancy networks positions tungsten Magnéli phases as key materials for emerging neuromorphic and ionotronic technologies. Controlled migration of oxygen vacancies under external electric fields allows reproducible resistance switching, enabling memristive behavior that mimics synaptic plasticity with low energy consumption and high endurance [92,93].

Finally, the intrinsic structural stability of Magnéli phases, arising from ordered crystallographic shear planes, enables robust operation under extreme conditions. These materials demonstrate remarkable resilience to thermal degradation, redox cycling, and harsh environments, making them promising candidates for high-temperature energy systems and advanced aerospace applications [40]. Overall, the multifunctionality of PLD-grown tungsten Magnéli phases originates from the unique interplay between non-equilibrium synthesis, defect engineering, and structural complexity, which together enable tunable electronic, ionic, and optical properties across a wide spectrum of advanced technologies.

## 7. Practical Scalability and High-Temperature Durability Challenges

From a technological and industrial perspective, translating the outstanding thermoelectric performance of pulsed laser deposition (PLD)-grown tungsten Magnéli phases into commercial devices requires addressing significant roadblocks related to processing scalability, manufacturing costs, and long-term high-temperature durability. It must be critically highlighted that PLD is inherently a high-vacuum, batch-oriented technique characterized by limited deposition areas and high capital equipment costs, making it suboptimal for large-scale industrial manufacturing. To bridge the gap between proof-of-concept laboratory thin films and broad commercial application, alternative high-throughput physical vapor deposition (PVD) techniques must be explored. Reactive magnetron sputtering and large-area plasma-enhanced chemical vapor deposition (PECVD) represent viable industrial pathways [51,94]. These techniques can replicate the high kinetic energy profiles and far-from-equilibrium thermodynamic conditions of PLD, facilitating the controlled injection of oxygen vacancies and the scalable nucleation of crystallographic shear planes over larger substrate areas. Beyond synthesis scalability, the operational durability of oxygen-deficient ${WO}_{x}$ thin films under continuous high-temperature environments poses a severe thermodynamic challenge. At elevated temperatures exceeding 600 K, sub-stoichiometric transition metal oxides are prone to intense oxidation and structural relaxation when exposed to even trace amounts of ambient oxygen [36,95]. The degradation mechanism typically involves spontaneous oxygen re-absorption, which progressively fills the engineered oxygen vacancies and destroys the sub-stoichiometric Magnéli shear planes. This structural relaxation transforms the highly conductive suboxide phase back into highly resistive, stoichiometric ${WO}_{3}$, leading to a catastrophic collapse of electrical conductivity and a failure of the thermoelectric figure of merit (ZT). To mitigate this severe operational degradation, the implementation of oxygen-impermeable encapsulation layers, such as atomic layer deposition (ALD)-grown ${Al}_{2}O_{3}$ or dense amorphous silicon-based coatings, has been widely proposed in the recent literature [96]. However, a critical evaluation of the current state of the art reveals a significant gap: there is a critical shortage of experimental literature reporting long-term, high-temperature cyclic testing for capped ${WO}_{x}$ systems. While encapsulation barriers effectively prevent oxygen ingress during short-term isothermal treatments, their long-term reliability under harsh thermal cycling conditions remains uncertain. Severe thermal cycling introduces significant interfacial stresses due to mismatches in thermal expansion coefficients between the ${WO}_{x}$ active layer, substrate, and encapsulation coating. Over extended operation, such cyclic stresses can promote crack initiation, delamination, and interfacial spallation. Similar degradation phenomena have been reported in ${WO}_{x}$ thin-film systems, where repeated cycling leads to defect accumulation and loss of structural and functional stability [97,98,99,100]. Once the structural integrity of the encapsulation is compromised, rapid oxygen diffusion ensues, driving immediate suboxide degradation. Consequently, future research must systematically prioritize rigorous, long-term thermal cycling and aging benchmarks under real-world temperature gradients to establish the true economic and operational viability of tungsten Magnéli phases in stable high-temperature oxide thermoelectrics.

## 8. Conclusions and Future Perspectives

Pulsed laser deposition (PLD) of sub-stoichiometric tungsten oxide ($WO_{3-x}$) thin films enables defect-driven thermoelectric decoupling by introducing coherent crystallographic shear (CS) planes. These structures, belonging to the homologous Magnéli series ($W_{n}O_{3n-1}$ and $W_{n}O_{3n-2}$), successfully bypass classical transport trade-offs. Decoupling electronic and phononic pathways elevates electrical conductivity ($\sigma >1000\;S\;{\mathrm{cm}}^{-1}$) while suppressing lattice thermal conductivity near its amorphous limit ($\kappa_{L}\approx 1.2–1.6\;W\;m^{-1}K^{-1}$), exploiting the capacity of PLD to kinetically trap non-equilibrium metastable phases. Despite high power factors achieved in the optimized $WO_{2.9}$ regime (∼160 μW m^−1^ K^−2^), future deployment depends on overcoming three critical bottlenecks. First, structural ambiguity must be resolved by mapping local vacancies to separate point-defect mechanics from multi-dimensional shear transport while tracking mixed valences ($W^{6+}/W^{5+}/W^{4+}$). Second, stoichiometry and thickness gradients induced by the highly directional plasma plume must be mitigated using plume-shaping geometries and continuous substrate rotation. Finally, high-temperature instability in ambient air requires the development of oxygen-impermeable encapsulation layers (e.g., $Al_{2}O_{3}$ or dense silicates) to prevent defect annealing during thermal cycling. In summary, mastering PLD-controlled transport decoupling unlocks a clean, efficient pathway toward sustainable oxide-based thermoelectrics for industrial waste heat recovery. To overcome these persistent manufacturing and stability challenges, future research paradigms must pivot toward a coordinated co-design strategy that integrates precise plume dynamics with multi-scale protective coating architectures. By mastering the real-time fluid dynamic expansion of the ablation species through automated, feedback-controlled gas delivery systems and adaptive substrate positioning, it will be possible to eliminate the macroscopic stoichiometry and thickness variations that typically plague large-area pulsed laser deposition processing. Furthermore, engineering resilient, multi-layered encapsulation configurations, such as alternating atomic-layer-deposited aluminum oxide and dense silicate barrier stacks with tailored coefficients of thermal expansion, represents a definitive pathway to safeguard the fragile under-coordinated tungsten ions. Once these oxygen-impermeable encapsulation layers successfully mitigate atmospheric oxidation, appropriately protected tungsten Magnéli thin films can permanently isolate their high-mobility, edge-sharing octahedral channels, unlocking stable, degradation-free performance and enabling these binary sub-oxides to realistically achieve their highly efficient peak thermoelectric target values at extreme operating temperatures.

## Figures and Tables

**Table 1:** Summary of reported processing conditions, thermoelectric transport parameters (σ, *S*, κ), and other physical properties for various $WO_{x}$ and Magnéli phases from literature. N.R. denotes data not reported in the original reference.

Phase/Composition	$P_{O_{2}}$ (Pa)	$T_{\mathrm{sub}}$ (K)	σ ($S\cdot {\mathrm{cm}}^{-1}$)	*S* (μV·K^−1^)	κ ($W\cdot m^{-1}\cdot K^{-1}$)	Other Physical Parameters	Ref.
Amorphous $WO_{2.511}$–$WO_{2.982}$	0.01–2.8	295	$5\times 10^{-5}$–333	−40 to −220	0.9–1.6	—	[58]
Monoclinic $W_{18}O_{49}$	0.6	873	≈75	N.R.	N.R.	$E_{g}=2.65$ eV	[60]
Predominantly tetragonal $WO_{3}$	2	773	N.R.	N.R.	N.R.	$E_{g}$ = 3.06–3.47 eV;	[61]
(orthorhombic + tetragonal for 250 nm)						$E_{a}=0.14$–0.24 eV	
Amorphous $WO_{3}$ to	2.7–8.0	295–873	≈0.11–1.7 $\times \;10^{-3}$	N.R.	N.R.	$E_{g}=2.89$–3.42 eV	[60,62,63]
orthorhombic $WO_{3}$							
$WO_{x}$	3–10	295–473	N.R.	N.R.	N.R.	$A_{D}=3.25\times 10^{12}$–	[64]
						$1.24\times 10^{14}$ ${\mathrm{eV}}^{-1}{\mathrm{cm}}^{-3}$;	
						$E_{g}=3.12$–3.29 eV	
Monoclinic $WO_{3}$	10	873	N.R.	N.R.	N.R.	Coherence length =	[65]
						20–35 nm	
Orthorhombic $WO_{3}$	12	873	$1.2\times 10^{-3}$	N.R.	N.R.	$E_{g}=3.24$ eV;	[60]
						$E_{a}=0.215$ eV	
Substoichiometric $WO_{x}$	10–100	295–773	N.R.	N.R.	N.R.	Nanoparticle size	[66]
						∼20 nm	

**Figure A1:**
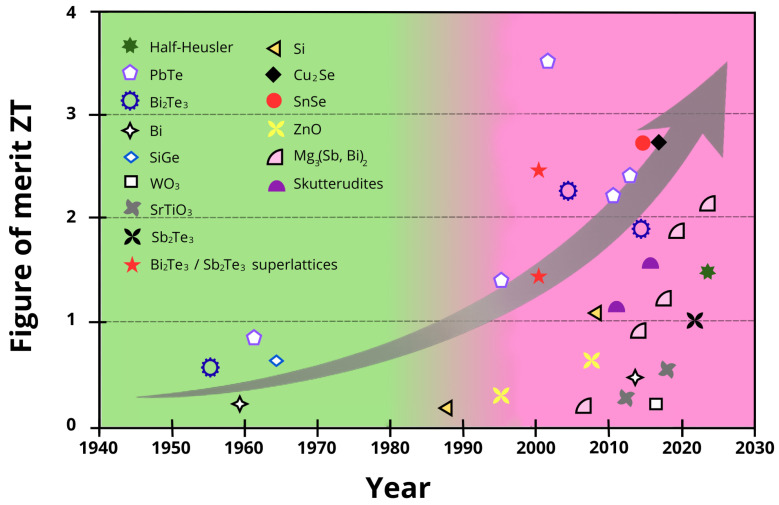
Evolution of the thermoelectric figure of merit (ZT) of representative materials from 1940. ZT values are reported at different temperatures depending on the material.

**Figure A2:**
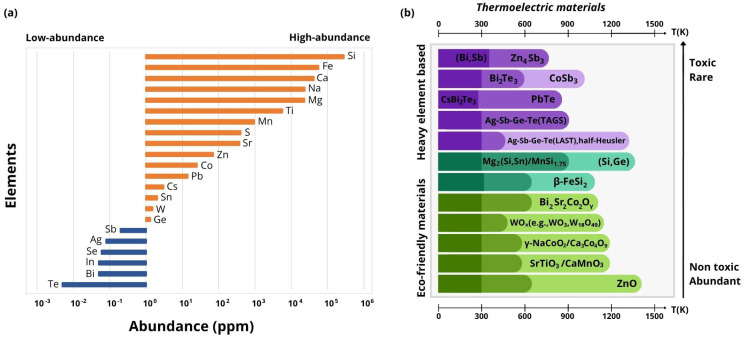
(**a**) Abundance of elements commonly used in thermoelectric materials, plotted on a logarithmic scale and expressed in parts per million (ppm). (**b**) Schematic comparison of selected thermoelectric (TE) materials, showing their operating temperature ranges and the abundance and environmental sustainability of their constituent elements.

**Figure A3:**
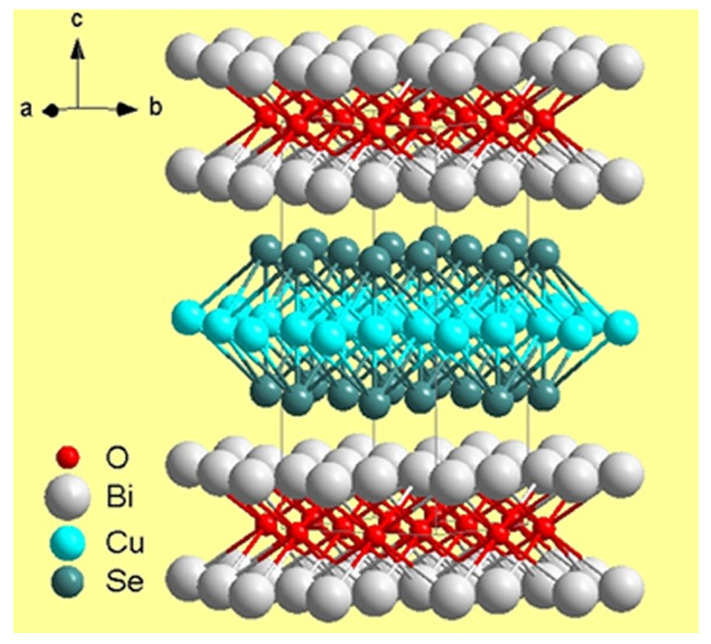
BiCuSeO layered oxides with P4/nmm space group. Figure adapted from Ref. [140] under the terms of the Creative Commons Attribution 4.0 International License.

**Figure A4:**
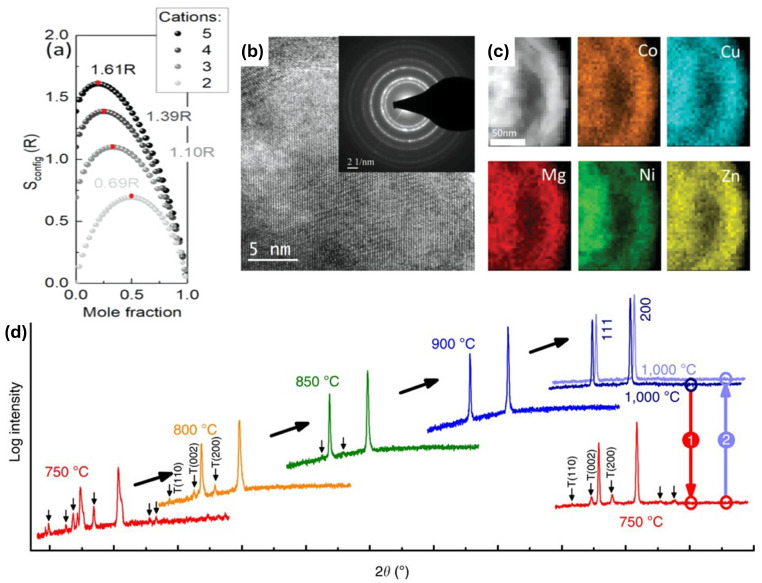
(**a**) Dependence of configurational entropy on the number of elements. (**b**) HR-TEM image and SAED pattern of R-HEO. (**c**) STEM image and elemental distribution maps indicating chemical uniformity of R-HEO. (**d**) Reversible phase transformation as an indication of entropy stabilization effect. Figures reproduced from Refs. [141,142] under the terms of the CC BY-NC-ND 4.0 and CC-BY 4.0 license, respectively.

## Data Availability

No new data were created or analyzed in this study. Data sharing is not applicable to this article.

## Abbreviations

The following abbreviations are used in this manuscript:

PLD	Pulsed Laser Deposition
ZT	Figure of Merit
HEO	High Entropy Oxide
2DEG	Two-Dimensional Electron Gas
DOS	Density Of States
HEO	High-Entropy Oxide
PGEC	Phonon-Glass Electron-Crystal
CS	Crystallographic Shear
PF	Power Factor

## Appendix A. Comparative Benchmarking with Non-Oxide Thermoelectric Systems

To provide a comprehensive benchmark for the decoupling strategies discussed for $WO_{x}$, these appendices review how similar transport mechanisms are tackled in high-performing non-oxide, high-entropy alloy systems (Appendix A) and other transition metal oxide (Appendix B).

Thermoelectric materials represent a cornerstone of state-of-the-art energy conversion technologies, converting heat directly into electricity via the Seebeck effect, where the voltage ΔV, generated across a temperature gradient ΔT, is given by ΔV=SΔT with *S* as the Seebeck coefficient (typically 50–400 μV/K for leading materials). However, device performance hinges on the dimensionless figure of merit: $ZT=\frac{S^{2}\sigma T}{\kappa }$ where σ is the electrical conductivity, κ is the thermal conductivity (due to the electronic $\kappa_{e}$ and reticular $\kappa_{L}$ components, respectively, and *T* is the absolute temperature [101,102,103,104]. A high ZT value requires a simultaneous optimization of three different transport properties: a large Seebeck coefficient, high electrical conductivity, and low thermal conductivity [105]. However, these parameters are strongly coupled, causing an intrinsic trade-off that makes their independent optimization very difficult. In particular, the Seebeck coefficient and electrical conductivity tend to exhibit opposite behaviors: materials with a high Seebeck coefficient, such as insulators or semiconductors with low carrier concentration, typically show poor electrical conductivity, whereas metals display high electrical conductivity but low Seebeck coefficients [106]. Consequently, the development of efficient thermoelectric materials represents a multiparametric optimization problem. Current research focuses on new strategies to partially decouple these properties, such as band structure engineering, nanostructuring and phonon scattering control [107,108].

Thermoelectric materials can be classified into inorganic and organic systems, each characterized by distinct advantages [109]. Inorganic materials, including chalcogenides, oxides and intermetallic compounds, typically exhibit higher ZT values and electrical conductivity, making them suitable for high-performance applications such as waste heat recovery (WHR) and large-scale energy conversion [110,111]. In contrast, organic materials, such as conductive polymers and carbon-based compounds, offer advantages in terms of flexibility, low density, and ease of processing [112]. These properties make them attractive for flexible devices, although their lower electrical conductivity generally results in reduced thermoelectric performance. This contrast emphasizes a fundamental trade-off in thermoelectric material design: inorganic materials offer high performance and efficiency, while organic materials provide flexibility and adaptability. Consequently, current research increasingly focuses on hybrid and composite systems that aim to combine these advantages [106]. Figure A1 displays the evolution of the thermoelectric figure of merit (ZT) of representative materials over time, highlighting the main research trends over the past decades. Early materials such as $Bi_{2}Te_{3}$, PbTe and SiGe, developed after the semiconductor revolution, typically exhibited ZT values close to unity and enabled the first practical applications. However, achieving ZT>2 remains essential for large-scale deployment. More recently, $Mg_{3}{\left(Sb,Bi\right)}_{2}$ alloys have emerged as promising low-cost alternatives [113], while oxide-based materials and $WO_{3}$ reflect the growing interest in sustainable thermoelectric systems, although their performance remains comparatively lower at present [3,106,114].

Building these considerations, the availability of constituent elements represents a critical factor for the future development of thermoelectric technologies. As shown in Figure A2a, many high-performance thermoelectric materials rely on relatively scarce elements, often with abundances below 1 ppm, raising concerns regarding cost, resource availability, and long-term sustainability. This is particularly evident for widely used chalcogenide-based systems such as $Bi_{2}Te_{3}$, PbTe, and related compounds [115]. At the same time, as illustrated in Figure A2b, these materials are typically employed within specific temperature ranges, with heavy-element-based compounds dominating low- and mid-temperature applications due to their superior performance [116].

In contrast, materials composed of earth-abundant and non-toxic elements, such as silicides and oxides, are generally more suitable for higher-temperature operation and offer improved chemical and thermal stability. In particular, oxide-based thermoelectric materials can operate under large temperature gradients in air, which may partially compensate for their lower ZT values by enabling higher conversion efficiencies [2,117]. Furthermore, their chemical versatility and low cost make them especially attractive for large-scale and sustainable applications. The current status of top-performing materials and their associated trade-offs are systematically summarized in Table A1.

**Table A1:** Comparison of representative thermoelectric materials. Reported Seebeck coefficients and peak ZT values refer to optimized systems (e.g., doped, nanostructured, or engineered compositions). For skutterudites, values correspond to filled $CoSb_{3}$. Data compiled from representative literature sources [118,119,120,121,122].

Materials	Seebeck Coefficient (μV/K)	ZT (Peak)	Key Advantages	Key Disadvantages
$Bi_{2}Te_{3}$ (Bulk)	150–250	∼1.0–1.5 (300 K)	High σ, low κ	Toxic, brittle, Te scarcity
PbTe	100–200	∼1.0–2.1 (600–800 K)	Highly tunable, nanostructurable	Pb toxicity, thermal instability
$CoSb_{3}$ (filled)	100–200	∼1.0–1.7 (600–800 K)	Low κ via filler atoms	Costly, oxidation sensitivity
SiGe	50–150	∼0.7–1.0 (900–1100 K)	High-temperature robustness	High κ, expensive processing
Oxide TE (bulk)	100–200	∼0.2–0.8 (700–1000 K)	Chemically stable, non-toxic	Low σ, lower efficiency

Bismuth telluride ($Bi_{2}Te_{3}$) alloys excel near room temperature (peak ZT≃ 1.0–1.5 below 450 K) but suffer from Te scarcity, brittleness, and rapid degradation above 200 °C. Lead telluride (PbTe) operates in the mid-temperature range (500–800 K, achieving ZT≃ 1.5–2.2 via nanostructuring) but is plagued by Pb toxicity and chemical instability. Filled skutterudites ($CoSb_{3}$)-based) approach ZT≃ 1.4–1.7 by realizing the ’phonon-glass electron-crystal’ (PGEC) paradigm, yet remain restricted by oxidation sensitivity and high processing costs. At high temperatures (>800 K), silicon-germanium (SiGe) alloys provide robust oxidation resistance (ZT≃ 0.8–1.2) but are limited by the high cost of *Ge* and a relatively high $\kappa_{L}$. Finally, state-of-the-art oxide perovskites (e.g., $Ca_{3}Co_{4}O_{9}$) and unoptimized oxides exhibit environmental benignity and stability, but their low power factors ($S^{2}\sigma$) yield modest ZT values (≃0.4–0.8) [106,123,124,125,126]. This multi-material cross-examination underscores the urgent need for novel transport-decoupling mechanisms—such as the defect-driven architectures explored in this review—to elevate oxide-based thermoelectrics to commercial viability.

## Appendix B. Transition Metal Oxides: Emerging Potential and Transport Decoupling Mechanisms

Conventional non-oxide thermoelectrics hold room-temperature figure of merit (ZT) records but suffer from poor high-temperature thermal stability, high toxicity, oxidation susceptibility, and reliance on scarce, expensive elements. Conversely, transition metal oxides offer unmatched operational advantages: stability exceeding 800 °C in ambient air, environmental sustainability, high mechanical strength, and chemical resistance to corrosive environments [116,127,128,129]. The performance efficiency of these thermoelectric materials is governed by the dimensionless expression: $ZT=\frac{S^{2}\sigma T}{\kappa_{e}+\kappa_{L}}$ where *S* is the Seebeck coefficient, σ is the electrical conductivity, *T* is the absolute temperature, and $\kappa_{e}$ and $\kappa_{L}$ are the electronic and lattice thermal conductivities, respectively. Intrinsic transport trade-offs pose a fundamental challenge rooted in solid-state physics. According to the Mott relation, *S* is inversely proportional to carrier concentration *n*, whereas σ scales linearly with it (σ=neμ,whereμiscarriermobility). Furthermore, $\kappa_{e}$ couples to σ via the Wiedemann–Franz law ($\kappa_{e}=L\sigma T,\;\mathrm{with}\;L\;\mathrm{being}\;\mathrm{the}\;\mathrm{Lorenz}\;\mathrm{number}$). Traditional optimization centered on aliovalent doping and mass-fluctuation scattering in intermetallics systematically fails in oxides due to fundamentally different physics. Instead of quasi-free electron transport in single parabolic bands, oxides feature narrow d-electron bands and intense electron–phonon coupling that trigger small-polaron formation and thermally activated hopping conduction. Consequently, standard doping induces deep intra-gap states that immobilize charge carriers, while traditional mass-disorder engineering degrades electronic mobility without lowering $\kappa_{L}$, which is already constrained by rigid ionic–covalent frameworks near the amorphous limit [130,131,132]. Breaking these rigid constraints requires mechanism-driven decoupling strategies that maximize the power factor ($\mathrm{PF}=S^{2}\sigma$) while independently suppressing $\kappa_{L}$, effectively treating the material as a “phonon-glass electron-crystal” (PGEC). For example, n-type ${La}_{0.5}{Na}_{0.5}{TiO}_{3}$ exploits A-site compositional disorder to disrupt thermal transport without degrading electronic conduction. Similarly, layered ${La}_{2}{CoO}_{4}$ achieves PGEC behavior by spatially isolating transport functions: conductive, perovskite-like ${LaCoO}_{3}$ layers act as electron crystals, whereas insulating LaO rock-salt layers serve as phonon glasses, reducing $\kappa_{L}$ via interfacial scattering. Layered structures inherently maximize ZT by decoupling σ (via polaron hopping or band conduction) from $\kappa_{L}$ through enhanced interface phonon scattering, as summarized for prominent p-type and n-type oxides in Table A2.

**Table A2:** Representative thermoelectric properties of p-type and n-type oxide materials. Reported values correspond to optimized compositions from the literature.

Material	Type	Seebeck (μV/K)	${\mathrm{ZT}}_{\max}$	σ (S/cm)	κ (W/mK)	Refs
$Ca_{3}Co_{4}O_{9}$ (film)	p	100–200	0.5–0.8 (900–1000 K)	20–200	0.8–1.5	[13,14]
$Na_{x}CoO_{2}$	p	70–120	0.8–1.2 (800 K)	$10^{3}$–$1.5\times 10^{4}$	1–2	[15,19]
BiCuSeO	p	200–350	1.1 (923 K)	50–300	0.4–0.8	[133]
$Bi_{2}Sr_{2}Co_{2}O_{y}$	p	100–150	0.15–0.20 (973 K)	50–200	1.5–2.5	[134]
$SrTiO_{3}$ (epitaxial)	n	−150–(−300)	0.20–0.30 (873 K)	100–4000	2–4	[17]
$SrTiO_{3}$ (2DEG)	n	−200–(−300)	∼1 (300 K)	$10^{3}$–$10^{4}$	2–4	[18]
$TiO_{2}:Nb$	n	−50–(−300)	0.10–0.18 (300–900 K)	25–1000	1.3–4.5	[16,135]
$WO_{x}$ (Magnéli)	n	−50–(−120)	0.07–0.15 (1100 K)	10–1000	1.0–2.0	[36,40,70]
ZnO:Al/Ga	n	−50–(−200)	0.47–0.65 (1000–1247 K)	500–1500	3–10	[136]
$CaMnO_{3}\;\;\left(Y/Nb\right)$	n	−120–(−300)	0.15–0.25 (950–1100 K)	100–400	1.2–2.0	[137,138]
$In_{2}O_{3}:Ge$	n	−100–(−250)	0.3–0.5 (1100–1273 K)	200–1200	1.5–3.0	[139]

Microstructural engineering further overcomes these transport limits. As summarized in Table A3 for p-type BiCuSeO oxyselenides (P4/nmm space group, composed of ${(Bi}_{2}O_{2}{)}^{2+}$ and ${(Cu}_{2}{Se}_{2}{)}^{2-}$ layers, Figure A3), point defects efficiently scatter short-wavelength phonons, while nano-grains suppress long-wavelength phonons. Combining both mechanisms enables full-spectrum phonon scattering, driving $\kappa_{L}$ toward its theoretical minimum.

**Table A3:** Effect of structural features on lattice thermal conductivity in BiCuSeO [133].

Analyzed Parameter	Key Finding	Lattice Thermal Conductivity ($\kappa_{L}$)
Pristine crystal value	Perfect BiCuSeO exhibits intrinsically ultra-low lattice thermal conductivity due to its layered structure	$\kappa_{L}\approx 0.84$ W m^−1^ K^−1^ at 300 K
Point defects	Bismuth (V_Bi_) and copper (V_Cu_) vacancies act as strong scattering centers for high-frequency phonons	Reduction in $\kappa_{L}$ by ∼14–21% compared to the pristine crystal
Nano-grains	Nanoscale grain boundaries (10–100 nm) effectively block low-frequency phonons with long mean free paths	Reduction in $\kappa_{L}$ by ∼40% in nanostructured samples
Combined synergistic effect	Simultaneous action of nano-grains and point defects enables full-spectrum phonon scattering	Suppression of $\kappa_{L}$ down to ∼0.4 W m^−1^ K^−1^ at 800 K

In strongly correlated cobaltites, spin-state transitions (low- to high-spin) introduce spin-entropy contributions that elevate *S* independently of *n*. Additionally, introducing nanoscopic potential barriers enables energy filtering, which selectively scatters low-energy carriers to increase *S* while preserving carrier mobility. Recent breakthroughs exploit entropy-driven structural stabilization, oxygen vacancy manipulation, and 2DEG confinement. High-entropy oxides (HEOs) utilize configurational entropy to stabilize single-phase crystalline structures containing five or more principal cations, generating severe local lattice distortions and mass-contrast fluctuations. In rock-salt HEOs (R-HEOs) like ${(Co}_{0.2}{Cu}_{0.2}{Mg}_{0.2}{Ni}_{0.2}{Zn}_{0.2})O$ (Figure 5a–c), this entropy-driven stabilization is demonstrated by temperature-dependent, reversible phase transformations (Figure A4). For instance, ${(Co}_{0.2}{Cu}_{0.2}{Mg}_{0.2}{Ni}_{0.2}{Zn}_{0.2})O$, ${(Ce}_{0.2}{Zr}_{0.2}{Hf}_{0.2}{Sn}_{0.2}{Ti}_{0.2}{)O}_{2}$, and ${(Gd}_{0.2}{La}_{0.2}{Nd}_{0.2}{Sm}_{0.2}Y_{0.2}{)MnO}_{3}$ transition to a multiphase mixture at lower temperatures but revert to a single-phase solid solution at higher temperatures (e.g., demixing at 750 °C and recovering at 1000 °C for R-HEO). This reversible, endothermic behavior proves that the $T\Delta S_{mix}$ term dominates over the enthalpy-driven phase separation. Conversely, HEOs like ${(Ce}_{0.2}{La}_{0.2}{Pr}_{0.2}{Sm}_{0.2}Y_{0.2}{)O}_{2-\delta }$ or ${(Gd}_{0.2}{La}_{0.2}{Nd}_{0.2}{Sm}_{0.2}Y_{0.2}{)FeO}_{3}$ lack this reversibility, as their single-phase lattice is already enthalpy-favorable ($\Delta H_{mix}<0$). Such structural irregularities act as full-spectrum phonon scattering centers; the extreme chemical disorder shortens the phonon mean free path to the interatomic distance (Ioffe–Regel limit). This neutralizes wave-like propagation into a localized, glass-like diffusion mode, enabling a temperature-independent, ultra-low thermal conductivity that preserves structural integrity under cyclic thermal loads. This neutralizes wave-like propagation into a localized, glass-like diffusion mode, enabling a temperature-independent, ultra-low thermal conductivity that preserves structural integrity under cyclic thermal loads.

To further minimize lattice thermal conductivity via the kinetic relation $\kappa_{L}=\frac{1}{3}C_{v}v_{g}\Lambda$ (where $C_{v}$ is heat capacity, $v_{g}$ is group velocity, and Λ is the phonon mean free path), explicit phononic scattering centers must be engineered. For instance, caged structures utilize weakly bound guest atoms (“rattlers”) whose low-frequency incoherent vibrations damp heat-carrying acoustic modes, while asymmetric bonding or lone pairs promote lattice anharmonicity and multi-phonon scattering. In oxide ceramics, manipulating the microstructure via grain boundaries and dense nanoscale interfaces establishes a panoscopic network: point defects scatter short-wavelength phonons, nanoprecipitates block mid-wavelengths, and grain boundaries target long-wavelength modes, driving $\kappa_{L}$ toward its theoretical minimum without compromising electron mobility. Crucially, tailoring these structural irregularities—such as crystallographic shear planes and oxygen vacancy networks—is dictated by the thermodynamic and kinetic conditions of the synthesis process. Top-down methods rely on severe lattice strain and macroscopic grain boundary engineering to lower $\kappa_{L}$, whereas bottom-up wet-chemical approaches provide atomic-scale precision for band structure manipulation. However, in highly reducible transition metal oxides like the ${WO}_{x}$ system, chemical synthesis often yields poorly ordered defect aggregates or excessive oxygen vacancy concentrations (>5–10%) that collapse S. Conversely, physical vapor deposition via magnetron sputtering optimizes film density and oxygen stoichiometry to enhance σ, but lacks the high kinetic energy window needed to precisely engineer ordered Magnéli phases and coherent planar defects. Exploiting ordered crystallographic shear zones for non-equilibrium phase stabilization strictly requires pulsed laser deposition (PLD) as the definitive tool to atomistically engineer defect-driven transport pathways [28,70,143,144,145,146].
